# Harmonising the human biobanking consent process: an Irish experience

**DOI:** 10.12688/hrbopenres.13384.1

**Published:** 2021-09-01

**Authors:** Lydia O'Sullivan, Tomás P. Carroll, Sarah Cooper, Ann Cullen, Laura Gorman, Billy McCann, Blánaid Mee, Nicola Miller, Verena Murphy, Máiréad Murray, Jackie O'Leary, Sharon O'Toole, Emma Snapes, Suzanne Bracken

**Affiliations:** 1 School of Medicine, University College Dublin, Dublin, D04 V1W8, Ireland; 2 Health Research Board-Trials Methodology Research Network, National University of Ireland Galway, Galway, H91 TK33, Ireland; 3 Alpha-1 Foundation Ireland, Royal College of Surgeons in Ireland, Education and Research Centre, Beaumont Hospital, Dublin, D09 YD60, Ireland; 4 Department of Gastroenterology, Hepatology and Nutrition, Children's Health Ireland at Crumlin, Dublin, D12 N512, Ireland; 5 Conway Institute, University College Dublin, Dublin, D04 V1W8, Ireland; 6 Department of Histopathology, St James’s Hospital, Dublin, D08 W9RT, Ireland; 7 Public and Patient Partner and member, National Research Ethics Committee, 67-72 Lower Mount Street, Dublin, D02 H638, Ireland; 8 National University of Ireland, Galway, Galway, H91 TK33, Ireland; 9 Cancer Trials Ireland, Innovation House, Glasnevin, Dublin, D11 KXN4, Ireland; 10 Irish Centre for Maternal and Child Health Research (INFANT), University College Cork, Cork, T12 YE02, Ireland; 11 Department of Obstetrics and Gynaecology/Histopathology, Trinity College Dublin, Trinity St James’s Cancer Institute, St James’s Hospital, Dublin, D08 W9RT, Ireland; 12 BioConsulting, Cork, Ireland; 13 Formerly of Clinical Research Development Ireland, 28 Mount Street Lower, Dublin, D02 CX28, Ireland

**Keywords:** Biobanking, Translational Research, Genetic Research, Clinical Research, Participant Information Leaflet, Informed Consent Form, Public and Patient Involvement, Data Protection.

## Abstract

Biobanks are repositories of human biological samples and data. They are an important component of clinical research in many disease areas and often represent the first step toward innovative treatments. For biobanks to operate, researchers need human participants to give their samples and associated health data. In Ireland, research participants must provide their freely given informed consent for their samples and data to be taken and used for research purposes. Biobank staff are responsible for communicating the relevant information to participants prior to obtaining their consent, and this communication process is supported by documentation in the form of Participant Information Leaflets and Informed Consent Forms (PILs/ICFs). PILs/ICFs should be concise, intelligible, and contain relevant information. While not a substitute for layperson and research staff discussions, PILs and ICFs ensure that a layperson has enough information to make an informed choice to participate or not. However, PILs/ICFs are often lengthy, contain technical language and can be complicated and onerous for a layperson to read. The introduction of the General Data Protection Regulation (GDPR) and the related Irish Health Research Regulation (HRR) presented additional challenges to the Irish biobank community. In May 2019, the National Biobanking Working Group (NBWG) was established in Ireland. It consists of members from diverse research backgrounds located in universities, hospitals and research centres across Ireland and a public/patient partner. The NBWG aimed to develop a suite of resources for health research biobanks via robust and meaningful patient engagement, which are accessible, GDPR/HRR-compliant and could be used nationally, including a PIL/ICF template. This open letter describes the process whereby this national biobank PIL/ICF template was produced. The development of this template included review by the Patient Voice in Cancer Research, led by Professor Amanda McCann at University College Dublin and the Health Research Data Protection Network.

## Abbreviations

EU: European Union; GDPR: General Data Protection Regulation; HRR: Health Research Regulation; HRDPN: Health Research-Data Protection Network; ICF: Informed Consent Form; NBWG: National Biobanking Working Group; PIL: Participant Information Leaflet; PVCR: Patient Voice in Cancer Research; REC: Research Ethics Committee

## Disclaimer

The views expressed in this article are those of the authors. Publication in HRB Open Research does not imply endorsement by the Health Research Board of Ireland.

## Background

In the context of health research, biobanks are managed repositories of human biological samples and associated health data, which are collected, stored and used to facilitate scientific and medical research ^1^. Biobanks are an important component of clinical research in many disease areas and often represent the first step toward innovative treatments ^2, 3^.

For biobanks to operate, researchers need human participants to voluntarily give their biological samples and relevant health data. In line with internationally accepted ethical standards in clinical research such as the Declaration of Helsinki ^4^, in Ireland, participants must provide their freely-given informed consent for these samples and data to be taken and used for research purposes ^5^. Research staff are responsible for communicating the relevant information to participants and ensuring participants understand the information they have been provided with before participants give their consent. This communication process is supported by Participant Information Leaflets/Informed Consent Forms (PILs/ICFs). PILs/ICFs should be concise ^6^, intelligible ^5^, and along with the conversation with the research staff, should give the relevant information so that a layperson can determine if they want to take part ^4^. However, clinical research PILs/ICFs are becoming longer ^7, 8^ and in the view of the authors of this letter, are not primarily written to meet the needs of a layperson. While the use of visuals to explain important or complex concepts is strongly recommended ^9– 11^, they are often under-utilised in clinical research PILs/ICFs ^12^. This was identified as a limitation of current biobank PIL/ICFs in use nationally. The Irish Health Research Regulations (HRR) ^5^ mandate suitable and specific measures for the processing of personal data for the purposes of health research in addition to the statutory obligations of the European Union (EU) General Data Protection Regulation (GDPR) ^13– 15^. These pieces of legislation have presented additional challenges to research biobankers who aspire to provide clear, concise information which supports the decision-making of potential research participants.

Biobank-based research is frequently investigative in nature, and therefore, the research aims are often broad. Biobank research increasingly yields genetic findings, the relevance of which may be unclear at the time of the analysis. These factors, among others, present additional challenges to ensure that participant consent is informed, while simultaneously ensuring that researchers have the freedom to explore and adapt their research as findings emerge, thus ensuring clinical research continues to advance, achieving the maximum possible information from the samples and data. Research staff are obliged and wish to provide information to participants in a clear and comprehendible way. However, research staff alone may not always be the best judge of what is understandable to the general public. Therefore, it is crucial that members of the general public and patients are involved in co-producing patient-facing documents, including clinical research PILs/ICFs. It can also be challenging for research ethics committees (RECs) and data protection officers to ensure that all of the required information is included in PILs/ICFs due to differing templates produced and wording favoured by individual institutions.

The biobank community came together in Ireland in early 2018, prior to the application of the GDPR legislation and a number of individuals subsequently volunteered to be part of the National Biobanking Working Group (NBWG). The NBWG was established in May 2019, originally under the auspices of Clinical Research Development Ireland. The group consists of members from diverse research backgrounds who are located in universities, hospitals and research centres across Ireland, and a public/patient partner. Each member has a special interest in making information about health research biobanks accessible and understandable to members of the public. The NBWG was established at a crucial juncture in the Irish health research landscape just after the implementation of new EU data protection legislation (GDPR) ^15^, Irish health research legislation (HHR) ^5^ and also the publication of the first international biobanking standard International Organization for Standardization (ISO) 20387: 2018 ^16^. The main aim of the NBWG was to address foreseen challenges for compliance brought about by the new legislation and jointly work towards an improved understanding of the reshaping of the biobank landscape in Ireland.

The NBWG has developed a suite of tools and resources, including infographics, a video, a general biobanking awareness leaflet and single PIL/ICF template specifically designed for health research biobanks, via robust and meaningful public and patient engagement, which could be used nationally. The NBWG also seeks to engage with members of the public and patients to increase awareness about the value of taking part in research biobanks. The inclusive and unified approach taken by the NBWG removes the need for multiple biobanks within Ireland to develop their own resources separately, a process which can be time-consuming and costly.

The aim of this open letter is to describe the process whereby a national template for a biobank PIL/ICF was co-produced with public-patient partners.

## Development of the PIL/ICF template

The NBWG initially convened via teleconference in May 2019. One significant challenge raised during this inaugural meeting was the lack of a standardized PIL/ICF template that could be used by all biobanks throughout Ireland. As informed consent is the cornerstone of all research, including biobanking, the group decided to prioritize this development.

To undertake this task, it was determined that the NBWG would meet on a bimonthly or monthly basis, initially in person, and subsequently via videoconference due to COVID-19 pandemic restrictions. As a starting point, the group collated and reviewed more than eight RECs-approved biobank PIL/ICF templates in use at that time across Irish universities and hospitals. A PIL/ICF developed by Cancer Trials Ireland, the leading cancer research organisation in Ireland was also reviewed ^17^. Based on the review process, it was agreed to divide the template PIL into two sections deemed most important (explained below). The group then reviewed and amended each section, using an iterative process, to ensure that the resulting template was applicable to a broad range of biobank research, disease areas and research environments (hospitals, academic institutions, not for profit organisations etc) within Ireland. The two sections were as follows:

1. ***Section 1: Joining a health research biobank*** – this section explains what a health research biobank is and invites individuals to take part. This section also outlines that participation is voluntary, that consent can be withdrawn, the benefits and risks of taking part, the procedure for biobanking and what will happen to participants’ health data should they choose to take part. The NBWG public/patient partner was particularly passionate about giving the most practical information at the start of the document, with further detail relevant to data protection to follow.
2. ***Section 2: What does the biobank do with your healthcare data?*** – this section outlines what kind of data will be collected and stored, the rationale for this and the participants’ rights. This section also outlines the security and compliance measures in place for participants’ data, details of the biobank funding, and ethics committee approval.

These two sections of the PIL are followed by the ICF, which is divided into two sections that correspond with the sections in the PIL (as described above).

Best practice guidelines for communicating clearly with laypersons, including the National Adult Literacy Agency (NALA) guidelines ^18^ were incorporated. For example, sentences were written in the active voice; plain, commonly used words were included whenever possible, and the section headings were posed as conversational-style questions to prompt pre-processing of information. Guidelines for optimal layout were also incorporated, such as using adequate line spacing and font size. Bold font was used for section headings as it is more accessible for dyslexic readers and those with literacy challenges, rather than underlining and all-capitals ^19– 21^. The group valued input from all members, but particularly from our public-patient partner to ensure that the language and content was understandable and relevant to research participants. The group informally shared the PIL/ICF with a selection of friends and family members who are neither medical professionals nor currently attending a hospital. It is important that those without regular medical contact or a previous diagnosis can understand the PIL/ICF, as individuals are often presented with the option of joining a biobank prior to confirmation of disease diagnosis.

**Figure 1.**
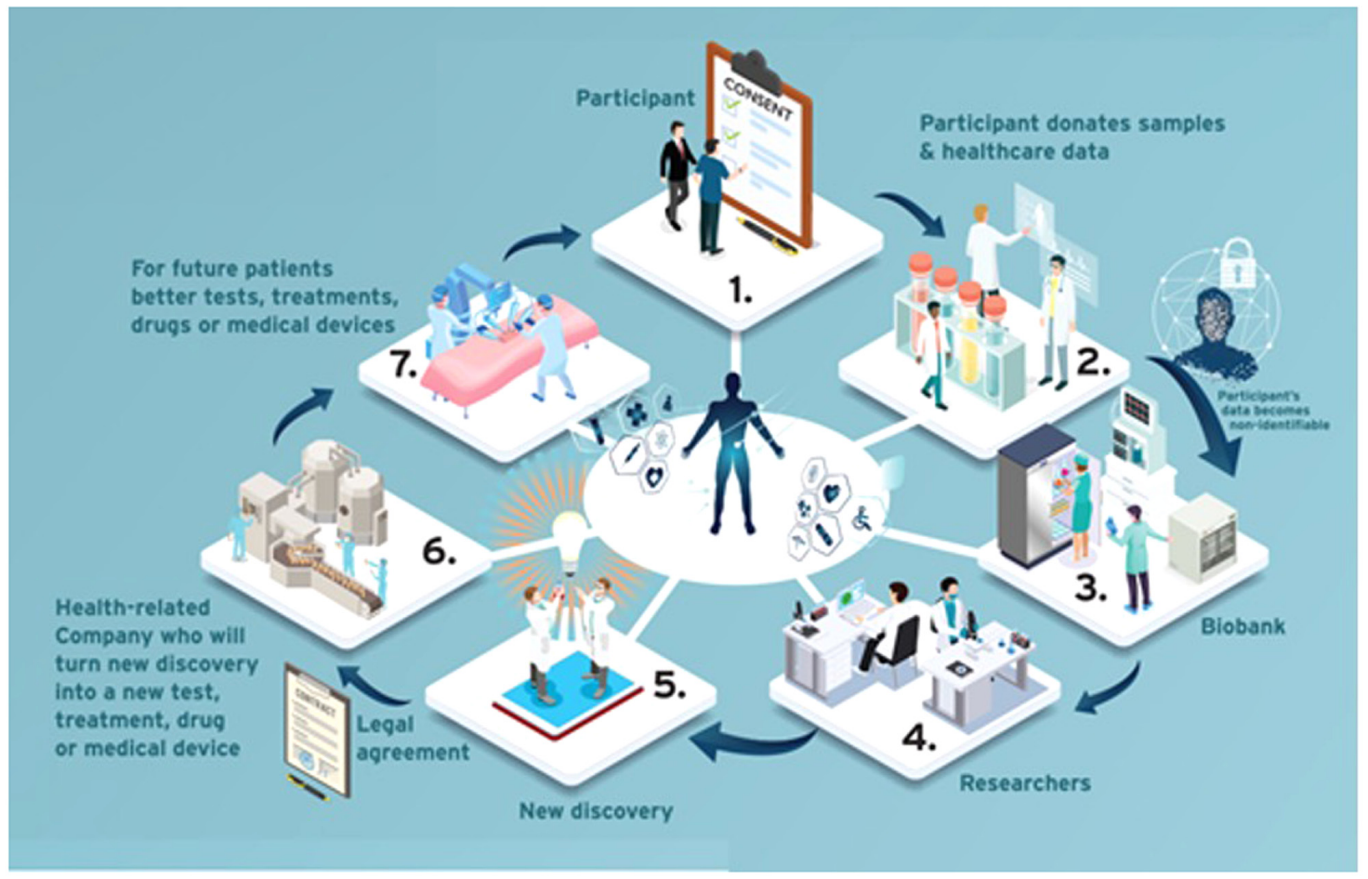
Infographic from the PIL/ICF template – *How a biobank can help future patients*

The group decided to include a glossary of key terms at the beginning of the PIL/ICF template to which readers could refer. Terms which laypersons may not be familiar with, such as ‘coded data’ and ‘identifiable data’ were included. To assess how well the template worked, the group decided to apply it to a well-established biobank at St James’s Hospital, Dublin; biobank-specific and site-specific information was added. Once the provisional content was decided, the group invited a professional graphic designer to design an infographic (see Figure 1).

) to explain how a health research biobank works. The graphic designer is a graduate of the Irish Platform for Patient Organisations, Science and Industry (IPPOSI) Patient Education Programme ^22^. They created a graphic with the participant at the centre of biobanking “where their voice and perspective is being seen and used as a valuable resource for the biobank”. The graphic highlights benefits to the participant and the wider society. A larger infographic was also developed which included some relevant facts about biobanks and health research (see *Extended data:* Supplementary File 1 ^23^). 

## Evaluation of the draft PIL/ICF template

### Patient Voice in Cancer Research Workshop

The group felt it was critical that the understandability and usability of the PIL/ICF template be evaluated by a wider independent group of laypersons. The Patient Voice in Cancer Research (PVCR), led by Professor Amanda McCann at University College Dublin, is an initiative that positively impacts on cancer research and outcomes for patients by actively engaging cancer patients, cancer researchers and other interested parties (patient advocates, families, carers, healthcare professionals, policymakers and those with an interest in cancer research) in discussions and decision-making processes ^24^. The PVCR and the NBWG co-hosted a workshop in Cork on 9 ^th^ October 2019 to develop public input into the draft template. To facilitate a critical appraisal of the documents, the workshop attendees were assigned to nine different groups of approximately 10 people each. Round-table discussions on an assigned topic were led by experienced facilitators. The topics were as follows:

- Is the PIL/ICF easy to understand? (two groups were assigned this topic as this was the main purpose of the review)
- Would patients be happy to consent to all parts of the consent form?
- Is it understood why samples and data are stored for a long time period?
- Does the document explain why data/samples may be shared with researchers around the world?
- Is it clear why samples and data may be shared with commercial companies?
- Are patients interested in updates on projects supported by Biobank?
- There is no national agreement on how research results which may affect your health should be returned to you, how do you feel about this?
- Do you understand from this document what genetic research means? Do you have any concerns?

A report from the roundtable discussions was prepared by Ms Yvonne D’Arcy of Darmah Market Research (see *Extended data:* Supplementary File 2 ^23^). Overall, the event participants agreed that the PIL/ICF template was accessible and user-friendly, and that the glossary of key terms and image were extremely helpful. PVCR attendees also provided informative feedback on content highlighting where it could be improved, including:

- clarifying the meaning of some of the key terms.
- emphasising the benefits to society in the future.
- clearly stating security measures in place for participant data.
- keeping GDPR information concise.

There were mixed views on whether potential participants should be offered the option to consent to some aspects but not others and whether ongoing updates should be provided to participants. The feedback was incorporated into the draft template. 

### Review by the Health Research Data Protection Network and the Data Protection Commission

The Health Research-Data Protection Network (HRDPN) was established in December 2018 to harmonise the approach of data protection officers working in health research environments in Ireland. The PIL/ICF template developed by the NBWG was sent to the HRDPN in 2020 and the resulting feedback was discussed and the documents were amended per their feedback. The PIL/ICF template has also been submitted to the Data Protection Commission for their review and the group awaits their feedback.

The final template PIL/ICF is included as Supplementary File 3 ( *Extended Data* ^23^).

## Implementing the PIL/ICF template

Some research groups have adopted the template PIL/ICF and it has been submitted for approval to various local RECs. Established biobanks have also requested the documents for use at their institutions. The PIL/ICF template has received positive feedback from the biobank community and the patient advocacy community. We welcome requests for use and feedback from other researchers or patient groups.

The infographic and biobank awareness leaflet (see *Extended data:* Supplementary File 4 ^23^) are currently in use in Irish Cancer Society Daffodil Centres, several research centres and hospital clinics nationally.

## Limitations

There are some limitations to this project. The PIL/ICF template developed by this group is intended for adult readers with the capacity to consent. Therefore, additional and/or different considerations will be needed for PIL/ICFs for children or adults lacking the capacity to give their consent. However, the process for the design and evaluation of a PIL/ICF could easily be adapted to facilitate stakeholder engagement for children and vulnerable research participants. While this PIL/ICF template was designed with the health research biobank in mind, the overall structure and much of the content could be applied to other forms of health research.

## Future directions

The experience of the NBWG members is that the standard data protection information included in biobank PILs is often too complicated and poorly understood by research participants. For this reason, the group attempted to use easy-to-understand language and to explain terminology, which is not in common use. To receive feedback on this aspect, the group intends to submit the PIL/ICF to the NALA for a full review. In addition, the group hopes to receive feedback from the Data Protection Commission on the accuracy of the explanations of the data protection terminologies. Ongoing work is focused on the production of a video version of the PIL/ICF specifically aimed at making the information more accessible to individuals with literacy challenges. Finally, the group hopes that the newly-developed biobank PIL/ICF template will eventually be adopted nationally as a standardised template, with the option to accordingly adapt it for specific patient groups. Ideally the group would favour a national standardised suite of documents and are willing to engage with other public/ patient groups, including those representing minorities such as those with disabilities and members of the travelling community, to achieve this goal.

## Data availability

### Underlying data

No data are associated with this article.

### Extended data

Open Science Framework: Harmonising the human biobanking consent process: an Irish experience, https://doi.org/10.17605/OSF.IO/5M8FP ^23^.

This project contains the following extended data:

- Supplementary File 1: Infographic
- Supplementary File 2: Patient Voice in Cancer Research Workshop Report
- Supplementary File 3: Template information leaflet and consent form
- Supplementary File 4: Infographic and biobank awareness leaflet

Data are available under the terms of the Creative Commons Zero "No rights reserved" data waiver (CC0 1.0 Public domain dedication).
