# Harmonising the human biobanking consent process: an Irish experience

**DOI:** 10.12688/hrbopenres.13384.3

**Published:** 2022-01-13

**Authors:** Lydia O'Sullivan, Tomás P. Carroll, Niamh Clarke, Sarah Cooper, Ann Cullen, Laura Gorman, Billy McCann, Blánaid Mee, Nicola Miller, Verena Murphy, Máiréad Murray, Jackie O'Leary, Sharon O'Toole, Emma Snapes, Suzanne Bracken

**Affiliations:** 1 School of Medicine, University College Dublin, Dublin, D04 V1W8, Ireland; 2 Health Research Board-Trials Methodology Research Network, National University of Ireland Galway, Galway, H91 TK33, Ireland; 3 Alpha-1 Foundation Ireland, Royal College of Surgeons in Ireland, Education and Research Centre, Beaumont Hospital, Dublin, D09 YD60, Ireland; 4 The Irish Longitudinal Study on Ageing (TILDA), Trinity College Dublin, Dublin, D02 R590, Ireland; 5 Department of Gastroenterology, Hepatology and Nutrition, Children's Health Ireland at Crumlin, Dublin, D12 N512, Ireland; 6 Conway Institute, University College Dublin, Dublin, D04 V1W8, Ireland; 7 Department of Histopathology, St James’s Hospital, Dublin, D08 W9RT, Ireland; 8 Public and Patient Partner and member, National Research Ethics Committee, 67-72 Lower Mount Street, Dublin, D02 H638, Ireland; 9 National University of Ireland, Galway, Galway, H91 TK33, Ireland; 10 Cancer Trials Ireland, Innovation House, Glasnevin, Dublin, D11 KXN4, Ireland; 11 Irish Centre for Maternal and Child Health Research (INFANT), University College Cork, Cork, T12 YE02, Ireland; 12 Department of Obstetrics and Gynaecology/Histopathology, Trinity College Dublin, Trinity St James’s Cancer Institute, St James’s Hospital, Dublin, D08 W9RT, Ireland; 13 BioConsulting, Cork, Ireland; 14 Formerly of Clinical Research Development Ireland, 28 Mount Street Lower, Dublin, D02 CX28, Ireland

**Keywords:** Biobanking, Translational Research, Genetic Research, Clinical Research, Participant Information Leaflet, Informed Consent Form, Public and Patient Involvement, Data Protection.

## Abstract

Biobanks are repositories of human biological samples and data. They are an important component of clinical research in many disease areas and often represent the first step toward innovative treatments. For biobanks to operate, researchers need human participants to give their samples and associated health data. In Ireland, research participants must provide their freely given informed consent for their samples and data to be taken and used for research purposes. Biobank staff are responsible for communicating the relevant information to participants prior to obtaining their consent, and this communication process is supported by the Participant Information Leaflets and Informed Consent Form (PI/ICFs). PILs/ICFs should be concise, intelligible, and contain relevant information. While not a substitute for layperson and research staff discussions, PILs and ICFs ensure that a layperson has enough information to make an informed choice to participate or not. However, PILs/ICFs are often lengthy, contain technical language and can be complicated and onerous for a layperson to read. The introduction of the General Data Protection Regulation and the related Irish Health Research Regulation presented additional challenges to the Irish biobank community. In May 2019, the National Biobanking Working Group (NBWG) was established in Ireland. It consists of members from diverse research backgrounds located in universities, hospitals and research centres across Ireland and a public/patient partner. The NBWG aimed to develop a suite of resources for health research biobanks via robust and meaningful patient engagement, which are accessible, General Data Protection Regulation/Health Research Regulation-compliant and could be used nationally, including a PIL/ICF template. This open letter describes the process whereby this national biobank PIL/ICF template was produced. The development of this template included review by the Patient Voice in Cancer Research, led by Professor Amanda McCann at University College Dublin and the Health Research Data Protection Network.

## Abbreviations

ICF: Informed Consent Form; NBWG: National Biobanking Working Group; PIL: Participant Information Leaflet;

## Disclaimer

The views expressed in this article are those of the authors. Publication in HRB Open Research does not imply endorsement by the Health Research Board of Ireland.

## Background

In the context of health research, biobanks are managed repositories of human biological samples and associated health data, which are collected, stored and used to facilitate scientific and medical research ^1^. Biobanks are an important component of clinical research in many disease areas and often represent the first step toward innovative treatments ^2, 3^.

For biobanks to operate, researchers need human participants to voluntarily give their biological samples and relevant health data. In line with internationally accepted ethical standards in clinical research such as the Declaration of Helsinki ^4^, in Ireland, participants must provide their freely-given informed consent for these samples and data to be taken and used for research purposes ^5^. Research staff are responsible for communicating the relevant information to participants and ensuring participants understand the information they have been provided with before participants give their consent. This communication process is supported by Participant Information Leaflets/Informed Consent Forms (PILs/ICFs). PILs/ICFs should be concise ^6^, intelligible ^5^, and along with the conversation with the research staff, should give the relevant information so that a layperson can determine if they want to take part ^4^. (For the purposes of this article, we define laypersons to be members of the public who, due to their background, may not be familiar with aspects of the research process. These individuals may have a wealth of knowledge from their lived experience but are not generally familiar with e.g., research terminology, or the role of the health research biobank. This means that these individuals may find it difficult to understand written or verbal information if it is not simple and free of jargon.) However, clinical research PILs/ICFs are becoming longer ^7, 8^ and in the view of the authors of this letter, are not primarily written to meet the needs of a layperson. Systematic reviews have shown that clinical research participants often have not understood important aspects of a research study they have agreed to take part in 9, 10, particularly those with literacy challenges ^11^. While the use of visuals to explain important or complex concepts is strongly recommended ^12, 13^, they are often under-utilised in clinical research PILs/ICFs ^14^. This was identified as a limitation of current biobank PIL/ICFs in use nationally. The Irish Health Research Regulations ^5^ mandate suitable and specific measures for the processing of personal data for the purposes of health research in addition to the statutory obligations of the European Union General Data Protection Regulation ^15– 17^. These pieces of legislation have presented additional challenges to research biobankers who aspire to provide clear, concise information which supports the decision-making of potential research participants. For example, this legislation requires that additional information about data protection measures should be provided to research participants. While we welcome research participants being provided with all the pertinent information about their data protection rights, we have found that this additional information is often provided in a format which is not well understood by laypersons. For example, legal jargon is often used and the use of the passive voice, verb nominalisations etc, can make it difficult to understand. 

Biobank-based research is frequently investigative in nature, and therefore, the research aims are often broad. Biobank research increasingly yields genetic findings, the relevance of which may be unclear at the time of the analysis. These factors, among others, present additional challenges to ensure that participant consent is informed, while simultaneously ensuring that researchers have the freedom to explore and adapt their research as findings emerge, thus ensuring clinical research continues to advance, achieving the maximum possible information from the samples and data. Research staff are obliged and wish to provide information to participants in a clear and comprehensible way. However, research staff alone may not always be the best judge of what is understandable to the general public. Therefore, it is crucial that members of the general public and patients are involved in co-producing patient-facing documents, including clinical research PILs/ICFs ^18– 20^. It can also be challenging for research ethics committees and data protection officers to ensure that all of the required information is included in PILs/ICFs due to differing templates produced and wording favoured by individual institutions.

The biobank community in Ireland came together in early 2018, prior to the application of the General Data Protection Regulation legislation and several individuals subsequently volunteered to be part of the National Biobanking Working Group (NBWG). The NBWG was established in May 2019, originally under the auspices of Clinical Research Development Ireland. The group consists of members from diverse research backgrounds who are located in universities, hospitals and research centres across Ireland, and a public/patient partner. Each member has a special interest in making information about health research biobanks accessible and understandable to members of the public. Group members are involved in maternal, infant, paediatric, adult and older adult research areas. The public/patient member is a patient advocate who doesn’t have a scientific, medical or research background and is therefore ideally placed to represent the perspectives of laypersons. The NBWG was established at a crucial juncture in the Irish health research landscape just after the implementation of new European Union data protection legislation (General Data Protection Regulation) ^17^, Irish health research legislation (Health Research Regulations) ^5^ and also the publication of the first international biobanking standard International Organization for Standardization (ISO) 20387: 2018 ^21^. The main aim of the NBWG was to address foreseen challenges for compliance brought about by the new legislation and to jointly work towards an improved understanding of the reshaping of the biobank landscape in Ireland.

The NBWG has developed a suite of tools and resources, including infographics, a general biobanking awareness leaflet and single PIL/ICF template specifically designed for health research biobanks, via robust and meaningful public and patient engagement, which could be adopted nationally. The group also developed a video which describes some of the research at St James’ Hospital, Dublin and Trinity College Dublin: https://www.stjames.ie/cancer/research/biobanknetwork/ This video aims to increase awareness of the importance of health research biobanks, and has been shared widely on social media and is played in multiple waiting rooms in St James’ Hospital, Dublin. The NBWG also seeks to engage with members of the public and patients to increase awareness about the value of taking part in research biobanks. The inclusive and unified approach taken by the NBWG removes the need for multiple biobanks within Ireland to develop their own resources separately, a process which can be time-consuming and costly.

The aim of this open letter is to describe the process whereby a national template for a biobank PIL/ICF was co-produced by the NBWG, with public-patient partners from the Patient Voice in Cancer Research. While we recognise that regulatory and research governance requirements, and cultural perspectives will vary between countries, the process may be of interest to other countries seeking to develop a national template.

## Development of the PIL/ICF template

The NBWG initially convened via teleconference in May 2019. One significant challenge raised during this inaugural meeting was the lack of a standardized PIL/ICF template that could be used by all biobanks throughout Ireland. As informed consent is the cornerstone of all research, including biobanking, the group decided to prioritize this development.

To undertake this task, it was determined that the NBWG would meet on a bimonthly or monthly basis, initially in person, and subsequently via videoconference due to COVID-19 pandemic restrictions. As a starting point, the group collated and reviewed more than eight research ethics committee-approved biobank PIL/ICF templates in use at that time across Irish universities and hospitals. A PIL/ICF developed by Cancer Trials Ireland, the leading cancer research organisation in Ireland was also reviewed 22. Based on the review process, it was agreed to divide the template PIL into three sections deemed most important (explained below). The group then reviewed and amended each section, using an iterative process, ensuring that the resulting template was applicable to a broad range of biobank research, disease areas and research environments (hospitals, academic institutions, not for profit organisations etc) within Ireland. The NBWG’s public-patient member was involved in every stage of the development and review process and in particular advised on whether language was understandable and relevant to research participants. The three sections were as follows:

1. ***Section A: Taking part*** – This section explains what a health research biobank is and invites individuals to take part. It outlines that participation is voluntary, that consent can be withdrawn, the benefits and risks of taking part, the procedure for biobanking and what will happen to participants’ health data should they choose to take part. The NBWG public-patient member was particularly passionate about giving the most practical information at the start of the document, with further detail relevant to data protection to follow.
2. ***Section B: Biobank Management*** – This section describes the security and compliance measures in place for participants’ data, details of the biobank funding, and ethics committee approval.
3. ***Section C: What does the biobank do with my healthcare data?*** – This section outlines what kind of data will be collected and stored, the rationale for this and the participants’ rights.

These three sections of the PIL are followed by the ICF, which is divided into two sections.

Best practice guidelines for communicating clearly with laypersons, including the National Adult Literacy Agency (NALA) guidelines ^23^ were incorporated. For example, sentences were written in the active voice; plain, commonly used words were included whenever possible, and the section headings were posed as conversational-style questions to prompt pre-processing of information. Guidelines for optimal layout were also incorporated, such as using adequate line spacing and font size. Bold font was used for section headings as it is more accessible for dyslexic readers and those with literacy challenges, rather than underlining and all-capitals ^24– 26^. The group valued input from all members, but particularly from our public-patient member to ensure that the language and content was understandable and relevant to research participants. The group informally shared the PIL/ICF with a selection of friends and family members who are neither medical professionals nor currently attending a hospital. It is important that personnel without regular medical contact or a previous diagnosis can understand the PIL/ICF, as individuals are often presented with the option of joining a biobank prior to confirmation of disease diagnosis.

**Figure 1.**
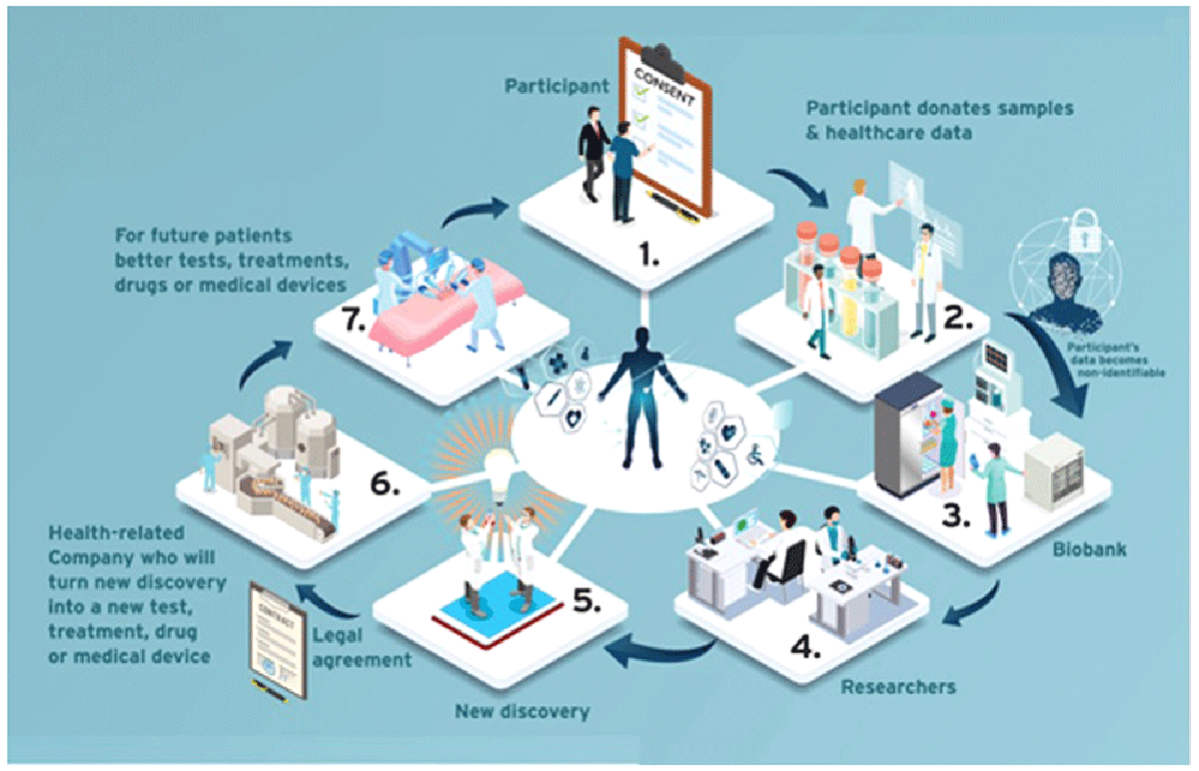
Infographic from the PIL/ICF template – *How a biobank can help future patients*.

The group decided to include a glossary of key terms at the beginning of the PIL/ICF template to which readers could refer. Terms which laypersons may not be familiar with, such as ‘coded data’ and ‘identifiable data’ were included. To assess how well the template worked, the group decided to apply it to a well-established biobank at St James’s Hospital, Dublin; biobank-specific and site-specific information was added. Once the provisional content was decided, the group invited a professional graphic designer to design an infographic (see Figure 1) ) to explain how a health research biobank works. The graphic designer is a graduate of the Irish Platform for Patient Organisations, Science and Industry (IPPOSI) Patient Education Programme ^27^. The graphic was created with the participant at the centre of biobanking and highlights the benefits to the participant and the wider society. A larger infographic was also developed which included some relevant facts about biobanks and health research (see *Extended data:* Supplementary File 1 ^28^.

## Evaluation of the draft PIL/ICF template

### Patient Voice in Cancer Research Workshop

The group felt it was critical that the understandability and usability of the PIL/ICF template be evaluated by a wider independent group of laypersons. The Patient Voice in Cancer Research, led by Professor Amanda McCann at University College Dublin, is an initiative that positively impacts on cancer research and outcomes for patients by actively engaging cancer patients, cancer researchers and other interested parties (patient advocates, families, carers, healthcare professionals, policymakers and those with an interest in cancer research) in discussions and decision-making processes ^29, 30^. The Patient Voice in Cancer Research and the NBWG co-hosted a workshop in Cork on 9 ^th^ October 2019 to develop public input into the draft template. To facilitate a critical appraisal of the documents, the workshop attendees were assigned to nine different groups of approximately 10 people each. Round-table discussions on an assigned topic were led by experienced facilitators. The topics were as follows:

- Is the PIL/ICF easy to understand? (two groups were assigned this topic as this was the main purpose of the review)
- Would patients be happy to consent to all parts of the consent form?
- Is it understood why samples and data are stored for a long time period?
- Does the document explain why data/samples may be shared with researchers around the world?
- Is it clear why samples and data may be shared with commercial companies?
- Are patients interested in updates on projects supported by the Biobank?
- There is no national agreement on how research results which may affect your health should be returned to you, how do you feel about this?
- Do you understand from this document what genetic research means? Do you have any concerns?

A report from the roundtable discussions was prepared by Ms Yvonne D’Arcy of Darmah Market Research (see *Extended data:* Supplementary File 2 ^28^). Overall, the event participants agreed that the PIL/ICF template was accessible and user-friendly, and that the glossary of key terms and images were extremely helpful. Patient Voice in Cancer Research attendees also provided informative feedback on content highlighting where it could be improved, including:

- clarifying the meaning of some of the key terms.
- emphasising the benefits to society in the future.
- clearly stating security measures in place for participant data.
- keeping General Data Protection Regulation information clear and concise.

There were mixed views on whether potential participants should be offered the option to consent to some aspects but not others and whether ongoing updates should be provided to participants. The feedback was incorporated into the draft template.

### Review by the Health Research Data Protection Network and the Data Protection Commission

The Health Research-Data Protection Network was established in December 2018 to harmonise the approach of data protection officers working in health research environments in Ireland. The PIL/ICF template developed by the NBWG was sent to the Health Research-Data Protection Network in 2020, the resulting feedback was discussed and the documents were amended per the feedback. The PIL/ICF template was also submitted to the Data Protection Commission for their review and the group awaits this feedback.

The final template PIL/ICF is included as Supplementary File 3 ( *Extended Data* ^28^).

## Implementing the PIL/ICF template

Some research groups have adopted the template PIL/ICF and it has been submitted for approval to various local research ethics committees. Established biobanks have also requested the documents for use at their institutions. The PIL/ICF template has received positive feedback from the biobank community and the patient advocacy community. We welcome requests for use and feedback from other researchers or patient groups.

The infographic and biobank awareness leaflet (see *Extended data:* Supplementary File 4 ^28^) are currently in use in Irish Cancer Society Daffodil Centres, several research centres and hospital clinics nationally.

## Limitations

There are some limitations to this project. The PIL/ICF template developed by this group is intended for adult readers with the capacity to consent. Therefore, additional and/or different considerations will be needed for PIL/ICFs for children or adults lacking the capacity to give their consent. However, the process for the design and evaluation of a PIL/ICF could easily be adapted to facilitate stakeholder engagement for children and vulnerable research participants. While this PIL/ICF template was designed with the health research biobank in mind, the overall structure and much of the content could be applied to other forms of health research.

## Future directions

The experience of the NBWG members is that the standard data protection information included in biobank PILs is often too complicated, lengthy and poorly understood by research participants. For this reason, the group attempted to use easy-to-understand language and to explain terminology, which is not in common use. To receive feedback on this aspect, the group intends to submit the PIL/ICF to the National Adult Literacy Agency for a full review. In addition, the group hopes to receive feedback from the Data Protection Commission on the accuracy of the explanations of the data protection terminologies. Ongoing work, using insights gained from the patient-public partners as part of this project, is focused on the production of a video version of the PIL/ICF specifically aimed at making the information more accessible to individuals with literacy challenges. The NBWG welcomes feedback from users of the PIL/ICF template and informal or formal evaluations of the template’s effectiveness. Finally, the group hopes that the newly-developed biobank PIL/ICF template will eventually be adopted nationally as a standardised template, with the option to accordingly adapt it for specific patient groups. Ideally the group would favour a national standardised suite of documents and are willing to engage with other public-patient groups, including those representing minorities such as those with disabilities and members of the travelling community, to achieve this goal.

## Data availability

### Underlying data

No data are associated with this article.

### Extended data

Open Science Framework: Harmonising the human biobanking consent process: an Irish experience, https://doi.org/10.17605/OSF.IO/5M8FP ^28^.

This project contains the following extended data:

- Supplementary File 1: Infographic
- Supplementary File 2: Patient Voice in Cancer Research Workshop Report
- Supplementary File 3: Template information leaflet and consent form
- Supplementary File 4: Infographic and biobank awareness leaflet

Data are available under the terms of the Creative Commons Zero "No rights reserved" data waiver (CC0 1.0 Public domain dedication).
