# Supplementary material for: Harmonising the human biobanking consent process: an Irish experience
Source: HRB Open Res. 2022 Jan 13;4:96. Originally published 2021 Sep 1. [Version 3] doi: 10.12688/hrbopenres.13384.3 (PMC8886168; doi:10.12688/hrbopenres.13384.3)
Supplement: Peer review history

# Peer review: Reviewer response for version 3

**DOI:** 10.21956/hrbopenres.14709.r31236

**Published:** 2022-02-28

**Authors:** Éidín Ní Shé, Ashfaq Chauhan, Laurel Mimmo

**Affiliations:** 1 Graduate School of Healthcare Management, RCSI University of Medicine and Health Sciences, Dublin, Ireland; 2 Australian Institute of Health Innovation, Macquarie University, Sydney, NSW, Australia; 3 School of Women’s and Children’s Health, University of New South Wales, Sydney, NSW, Australia; 4 Clinical Governance Unit, The Sydney Children’s Hospitals Network , Sydney, NSW, Australia

Thank you for this clarity and for providing the additional literature.

Does the article adequately reference differing views and opinions?

Partly

Are all factual statements correct, and are statements and arguments made adequately supported by citations?

Partly

Is the Open Letter written in accessible language?

Partly

Where applicable, are recommendations and next steps explained clearly for others to follow?

Yes

Is the rationale for the Open Letter provided in sufficient detail?

Yes

Reviewer Expertise:

Ashfaq Chauhan- Consumer engagement, co-design, patient safety, ethnic minorities Laurel Mimmo – pediatric healthcare quality and safety, consumer engagement, co-design, inclusive research, intellectual disability

We confirm that we have read this submission and believe that we have an appropriate level of expertise to confirm that it is of an acceptable scientific standard.

# Peer review: Reviewer response for version 3

**DOI:** 10.21956/hrbopenres.14709.r31237

**Published:** 2022-02-24

**Authors:** Nut Koonrungsesomboon

**Affiliations:** 1 Department of Pharmacology, Faculty of Medicine, Chiang Mai University, Chiang Mai, Thailand

I have one more suggestion for the authors: Figure 1 may be referred to in several presentations or used as a reference if it has a higher resolution.

Does the article adequately reference differing views and opinions?

Partly

Are all factual statements correct, and are statements and arguments made adequately supported by citations?

Partly

Is the Open Letter written in accessible language?

Yes

Where applicable, are recommendations and next steps explained clearly for others to follow?

Yes

Is the rationale for the Open Letter provided in sufficient detail?

Yes

Reviewer Expertise:

Human research ethics; Informed consent; Biobank research.

I confirm that I have read this submission and believe that I have an appropriate level of expertise to confirm that it is of an acceptable scientific standard.

# Peer review: Reviewer response for version 2

**DOI:** 10.21956/hrbopenres.14617.r30795

**Published:** 2021-11-22

**Authors:** Nut Koonrungsesomboon

**Affiliations:** 1 Department of Pharmacology, Faculty of Medicine, Chiang Mai University, Chiang Mai, Thailand

This open letter describes the developmental process of the national biobank informed consent template in Ireland. Overall, the manuscript is sufficient in general, however, there are some issues that need to be addressed.

1. The title of the two sections is not representative of the content provided therein. For example, “Joining a health research biobank” does not represent “the benefits and risks of taking part”. For another example, “details of the biobank funding” is not related to anything about “what does the biobank do with your healthcare data?” In addition, the content in the manuscript seems to be inconsistent with the template (in Supplementary File 3). In Supplementary File 3, there are 3 sections: Section A (Taking Part), Section B (Biobank Management), Section C (What does the biobank do with my healthcare data?)
2. In Figure 1, what is the meaning of the arrow from “7” to “1”? I do not understand the link between “For future patients better tests, treatments, drugs or medical devices” and “Participant”.
3. In Supplementary File 3 (the template), only the participant’s signature box is available. What if the participants cannot read and write? The authors should explain or discuss the implementation of the template as well. Whether or not the template is limited to those who can read, write, and sign consent. Or, whether or not the template must be modified to suit particular scenarios, and how to modify it?
4. The authors describe the general background about the biobank research and informed consent as well as the developmental process of the PIL/ICF template. However, the manuscript has lacked discussion on several issues. Are there any other templates in the world? What is the difference between the PIL/ICF template here and other templates? Pros and cons? Can the PIL/ICF template be applied outside Ireland? Can it be applied even outside Europe, like in the US or Asian countries? For example, in the US, there is the revised Common Rule that indicates the required elements of broad consent. Does the PIL / ICF template comply with the revised Common Rule? For another example, the CIOMS 2016 guidelines provide a guideline for the collection, storage, and use of biological materials and related data. Did the authors consider that guideline when developing the PIL/ICF template?

Does the article adequately reference differing views and opinions?

Partly

Are all factual statements correct, and are statements and arguments made adequately supported by citations?

Partly

Is the Open Letter written in accessible language?

Yes

Where applicable, are recommendations and next steps explained clearly for others to follow?

Yes

Is the rationale for the Open Letter provided in sufficient detail?

Yes

Reviewer Expertise:

Human research ethics; Informed consent; Biobank research.

I confirm that I have read this submission and believe that I have an appropriate level of expertise to confirm that it is of an acceptable scientific standard, however I have significant reservations, as outlined above.

# Response

**Published:** 2022-01-07

**Authors:** Lydia O'Sullivan

**Affiliations:** University College Dublin, Ireland

Thank you Prof Koonrungsesomboon for your time in reviewing our paper. We appreciate all of your comments and suggested changes. We have addressed each of the points you have raised and amended the paper accordingly.

 We have now amended the manuscript so that it matches the content in Supplementary File 3.

 The meaning of the arrow from “7” to “1” in Figure 1 relates to the fact that if a participant donates a biobank sample(s) and healthcare data, these can be used to develop new tests, treatments etc. These innovations will ultimately benefit research participants in the future. We explain this in the PIL (Supplementary File 3) within Section A/Point 3/What are the benefits to me? We also outline in this section that developing new tests, treatments etc often take a long time, so the benefits may not accrue to the participant themselves, but to others in the future. We recognise that no infographic/diagram will be able to explain the biobank process perfectly, but this version has been reviewed and approved by our wider public-patient partners. Our public-patient input highlighted how important it was to show a biobank as a circular process, rather then linear, which starts and ends with the participant. This is what we have tried to convey in this diagram. 

 Supplementary File 3/Part 1/Point 1 allows for participants who cannot read – “I have read, or had explained to me….”, but we do acknowledge the value of having a line for a witness to allow for participants who cannot write and we will consider adding this to a future version of the PIL/ICF template.

 We recognise that biobank PIL/ICFs templates exist in many other parts of the world. However, since the regulatory and research governance requirements, as well as cultural perspectives, vary in different countries and jurisdictions, we feel it is important that PIL/ICF templates are developed nationally or locally in conjunction with public-patient partners. The aim of this paper was to describe the experience we had in Ireland and we feel that researchers in other countries may be interested in this experience. We have produced a PIL/ICF template which aims to be compliant with the ethical and governance guidelines which are applicable in Ireland. We have now clarified this point at the end of the Background section, after stating the aim of the paper.

 Many thanks again.

# Peer review: Reviewer response for version 2

**DOI:** 10.21956/hrbopenres.14617.r30315

**Published:** 2021-09-17

**Authors:** Éidín Ní Shé, Ashfaq Chauhan, Laurel Mimmo

**Affiliations:** 1 Australian Institute of Health Innovation, Macquarie University, Sydney, NSW, Australia; 2 School of Women’s and Children’s Health, University of New South Wales, Sydney, NSW, Australia; 3 Clinical Governance Unit, The Sydney Children’s Hospitals Network , Sydney, NSW, Australia; 4 Graduate School of Healthcare Management, RCSI University of Medicine and Health Sciences, Dublin, Ireland

Thank you for the opportunity to read this open letter. We found this very interesting and consider it a significant issue as researchers working with people with intellectual disabilities and culturally and linguistically diverse populations in Australia.

 Please see below suggestions for you to consider:

1. Please provide some additional detail/examples for why the current PILs/ICFs are not suitable for layperson in the authors’ view, and any available feedback or evidence of what biobank participants want to know? For example, are the suitability issues related to length, content, and/or accessibility? Is there information missing from the current PILs/ICFs that biobank participants want or ask for, but is not included?
2. Please clarify who do you mean by layperson? Our population is inherently diverse and consists of many different groups including people who are refugees, are from diverse ethnic minority backgrounds or have specific communication needs.
3. Second paragraph: last line – what additional challenges do the regulations create? This can be briefly added to give reader some context. Are these additional challenges related to length, content, etc.?
4. Third paragraph – you have established some relevant links here e.g. importance of involving patient/public in developing patient-facing documents. This can be supported by references to academic literature.
5. While the effort taken by the authors to ensure the format of the information sheets met literacy guidelines is acknowledged, the NBWG membership has multiple representatives from various research fields, however, there is only a single public-patient partner, was this an individual person or a group of people? Please consider limitations that may be posed by lack of diverse patient/public representation in the working group as you have mentioned that only one member of the group was a patient/public. You could include some detail to highlight if they belonged to mainstream population group, or how they were representative of biobank participants, what level of experience they had and how this may have affected their contribution, and what level of actual contribution they made to the process and outcome.
6. The aim statement alludes that more than one patient/public partner was involved in the process while the above paragraph mentions one member of patient/public group as a member of the working group. Please clarify.
7. Thank you for describing the process in good detail. Were the patient/public partner also involved in drafting and generating ideas for the PIL/ICF draft and subsequent versions along with ensuring that the language was understandable and relevant to research participants. The workshop conducted to collect feedback on the draft is a great approach and authors may wish to consider commenting on the diversity of patient/public participants who provided feedback on the draft. 
8. In draft PILs (Section C, point 6), authors may consider adding a line that that staff can assist patient/participant to know their rights by providing them with a copy of the document if needed.
9. Is there a plan for evaluating the effectiveness of these revised forms? It would be prudent to see how they are working in practice, given the limited public-patient representation in the development. They could be tested with people from minority groups, such as those from ethnically diverse backgrounds, children and young people, and children and adults with intellectual disability. As there is limited detail on the involvement of diverse laypeople, the evaluation could include how useful the revised forms are for minority groups, and what adaptions may be necessary.
10. Infographic and use of multimedia are good but the letter lacks detail on whether they are being used by or helpful for the general public; what has been the feedback on these resources from biobank participants?
11. For question 4 our response is partly because there are many acronyms used. It is difficult to read a document with multiple acronyms, even where they are explained. We found we had to regularly go back and check some of the acronyms that are used only a few times in the letter. For example, GDPR, HRDPN, RECs. We would suggest acronyms are not used in these situations.

Does the article adequately reference differing views and opinions?

Partly

Are all factual statements correct, and are statements and arguments made adequately supported by citations?

Partly

Is the Open Letter written in accessible language?

Partly

Where applicable, are recommendations and next steps explained clearly for others to follow?

Yes

Is the rationale for the Open Letter provided in sufficient detail?

Yes

Reviewer Expertise:

Ashfaq Chauhan- Consumer engagement, co-design, patient safety, ethnic minorities Laurel Mimmo – pediatric healthcare quality and safety, consumer engagement, co-design, inclusive research, intellectual disability

We confirm that we have read this submission and believe that we have an appropriate level of expertise to confirm that it is of an acceptable scientific standard, however we have significant reservations, as outlined above.

# Response

**Published:** 2022-01-07

**Authors:** Lydia O'Sullivan

**Affiliations:** University College Dublin, Ireland

Thank you Mr Chauhan, Ms Mimmo and Dr Ní Shé, for your time in reviewing our paper. We appreciate all of your comments and suggested changes. We have addressed each of the points you have raised and amended the paper accordingly.

 We have now included additional references (References 9-11) and some text in the second paragraph of the Background section, explaining that the current clinical research PILs/ICFs are written at a level that is too complex – this means that the material is not accessible to many lay individuals, particularly those with literacy challenges. We have also included some references and text to note that systematic reviews have consistently demonstrated that clinical research participants, when assessed, frequently have an inadequate understanding of the research study they have agreed to take part in. We are not aware of any formal research which indicates that biobank participants feel that information is missing from PILs.

 We used the word ‘layperson’ to clarify that the intended audience for research PILs/ICFs are members of the public who, because of their background, may not be familiar with the research process. These individuals may have a wealth of knowledge from their lived experience but are not generally familiar with e.g., research terminology, or the role of the health research biobank. This means that these individuals may find it difficult to understand written or verbal information if it is not simple and free of jargon. Some of these individuals may be from diverse ethnic groups and have additional communication needs, as you have pointed out – the aim of our project was to create a PIL/ICF template which would be understandable to as many people as possible. We have now clarified our intended meaning of the word ‘layperson’ in the manuscript. 

 We have now added some sentences to clarify that the data protection regulations create additional challenges because they require additional information to be provided to research participants. While we welcome research participants being provided with the pertinent information about their data protection rights, we have found that this additional information is often provided in a format which is not well understand by an average member of the public. For example, legal jargon is often used and the structure of the sentences (e.g. use of the passive voice, verb nominalisations etc) makes it difficult to understand.

 We have now added some references (References 18-20) to the academic literature to describe the importance of involving patients/the public in developing patient-facing documents.

 One patient/public partner is a member of the National Biobank Working Group. This is noted in the following sentence in the *Background*/4 ^th^ paragraph: *The group consists of members from diverse research backgrounds who are located in universities, hospitals and research centres across Ireland, and a public/patient partner. *We have also added in the following sentence: *Group members are involved in maternal, infant, paediatric, adult and older adult research areas. *We have added in a sentence to describe this public/patient partner’s background and level of experience. However, the draft PIL/ICF template, once developed by the National Biobank Working Group, was reviewed and adapted, by a wider patient/public advocacy organisation – the Patient Voice in Cancer Research. This process, which is explained in the *Evaluation of the draft PIL/ICF template/Patient Voice in Cancer Research workshop* section, ensured that the template was reviewed by a larger group. The workshop was attended by approximately 90 people for 2 hours. The Patient Voice in Cancer Research group is composed of individuals from differing backgrounds including patients, family members, charities and the general public, all interested in cancer research. We have now included a reference to a paper that they have published (Reference 30), describing how they formed and how they operate. The NBWG is currently seeking more public-patient representatives to ensure a more balanced representation. 

 The patient/public partner was involved at every stage of drafting and generating ideas for the PIL/ICF draft and ensuring the language was understandable and relevant to research participants. We have now added a sentence in paragraph two of the ‘Development of the PIL/ICF template’ section to clarify this. As noted in point 5 above, we have now also added a reference (Reference 30) regarding the formation and composition of the patient/public participants at the workshop.

 We will certainly consider adding a sentence about staff providing a written copy of participant data protection rights in a future version the PIL/ICF template. We do also note, in several places in the template, that prospective participants can request further information from the investigator or study coordinator.

 This paper describes the first step in the process – co-developing a PIL/ICF template for biobanks with patient/public representatives. We acknowledge that it is important to evaluate the effectiveness of these form in practice and have now noted in the ‘ *Future Directions*’ section that we welcome such evaluations of effectiveness. We hope that this paper will increase awareness of this PIL/ICF template and lead to these kind of evaluations; however, an assessment of the effectiveness of the PIL/ICF template is outside  the scope of this paper. We disagree that the template development process involved ‘limited’ public-patient representation – as noted in point 5 above and as described in the paper, the template was reviewed and adapted following feedback from ~ 90 public-patient representatives, in addition to our NBWG patient representative who has been part of this work since its inception. This amounts to over 200 public-patient hours involvement in total. 

 Many thanks again.

# Response

**Published:** 2022-01-07

**Authors:** Lydia O'Sullivan

**Affiliations:** University College Dublin, Ireland

The aim of this open letter was to focus on the development of the PIL/ICF template, which includes a section of the infographic – both of these were reviewed by the Patient Voice in Cancer Research workshop. However, we have now noted that the knowledge gained from developing the PIL/ICF template was applied to preparing the video and a biobank awareness information leaflet. We have now also included more information about the video, which highlights the importance of biobank research. We acknowledge that getting feedback from biobank participants on the video is important, but it is outside of the scope of this paper.

 We have now removed the following acronyms from the manuscript:

-
  - EU: European Union
  - GDPR: General Data Protection Regulation
  - HRR: Health Research Regulation
  - HRDPN: Health Research-Data Protection Network
  - PVCR: Patient Voice in Cancer Research
  - REC: Research Ethics Committee
